# Infection with soil-transmitted helminths and their impact on coinfections

**DOI:** 10.3389/fpara.2023.1197956

**Published:** 2023-05-24

**Authors:** Josephine Schlosser-Brandenburg, Ankur Midha, Robert M. Mugo, Eric M. Ndombi, George Gachara, Doris Njomo, Sebastian Rausch, Susanne Hartmann

**Affiliations:** ^1^ Institute of Immunology, Centre for Infection Medicine, Freie Universität Berlin, Berlin, Germany; ^2^ Department of Medical Microbiology and Parasitology, Kenyatta University, Nairobi, Kenya; ^3^ Kenya Medical Research Institute, Centre for Global Health Research, Kisumu, Kenya; ^4^ Department of Medical Laboratory Science, Kenyatta University, Nairobi, Kenya; ^5^ Eastern and Southern Africa Centre of International Parasite Control, Kenya Medical Research Institute, Nairobi, Kenya

**Keywords:** *Ascaris*, *Trichuris*, hookworm, nematode, virus, bacteria, protozoa

## Abstract

The most important soil-transmitted helminths (STHs) affecting humans are roundworms, whipworms, and hookworms, with a large proportion of the world’s population infected with one or more of these intestinal parasites. On top of that, concurrent infections with several viruses, bacteria, protozoa, and other helminths such as trematodes are common in STH-endemic areas. STHs are potent immunomodulators, but knowledge about the effects of STH infection on the direction and extent of coinfections with other pathogens and *vice versa* is incomplete. By focusing on Kenya, a country where STH infections in humans are widespread, we provide an exemplary overview of the current prevalence of STH and co-occurring infections (e.g. with Human Immunodeficiency Virus, *Plasmodium falciparum*, *Giardia duodenalis* and *Schistosoma mansoni*). Using human data and complemented by experimental studies, we outline the immunomechanistic interactions of coinfections in both acutely STH transmigrated and chronically infected tissues, also highlighting their systemic nature. Depending on the coinfecting pathogen and immunological readout, STH infection may restrain, support, or even override the immune response to another pathogen. Furthermore, the timing of the particular infection and host susceptibility are decisive for the immunopathological consequences. Some examples demonstrated positive outcomes of STH coinfections, where the systemic effects of these helminths mitigate the damage caused by other pathogens. Nevertheless, the data available to date are rather unbalanced, as only a few studies have considered the effects of coinfection on the worm’s life cycle and associated host immunity. These interactions are complex and depend largely on the context and biology of the coinfection, which can act in either direction, both to the benefit and detriment of the infected host.

## 1 Introduction

**Figure 1:**
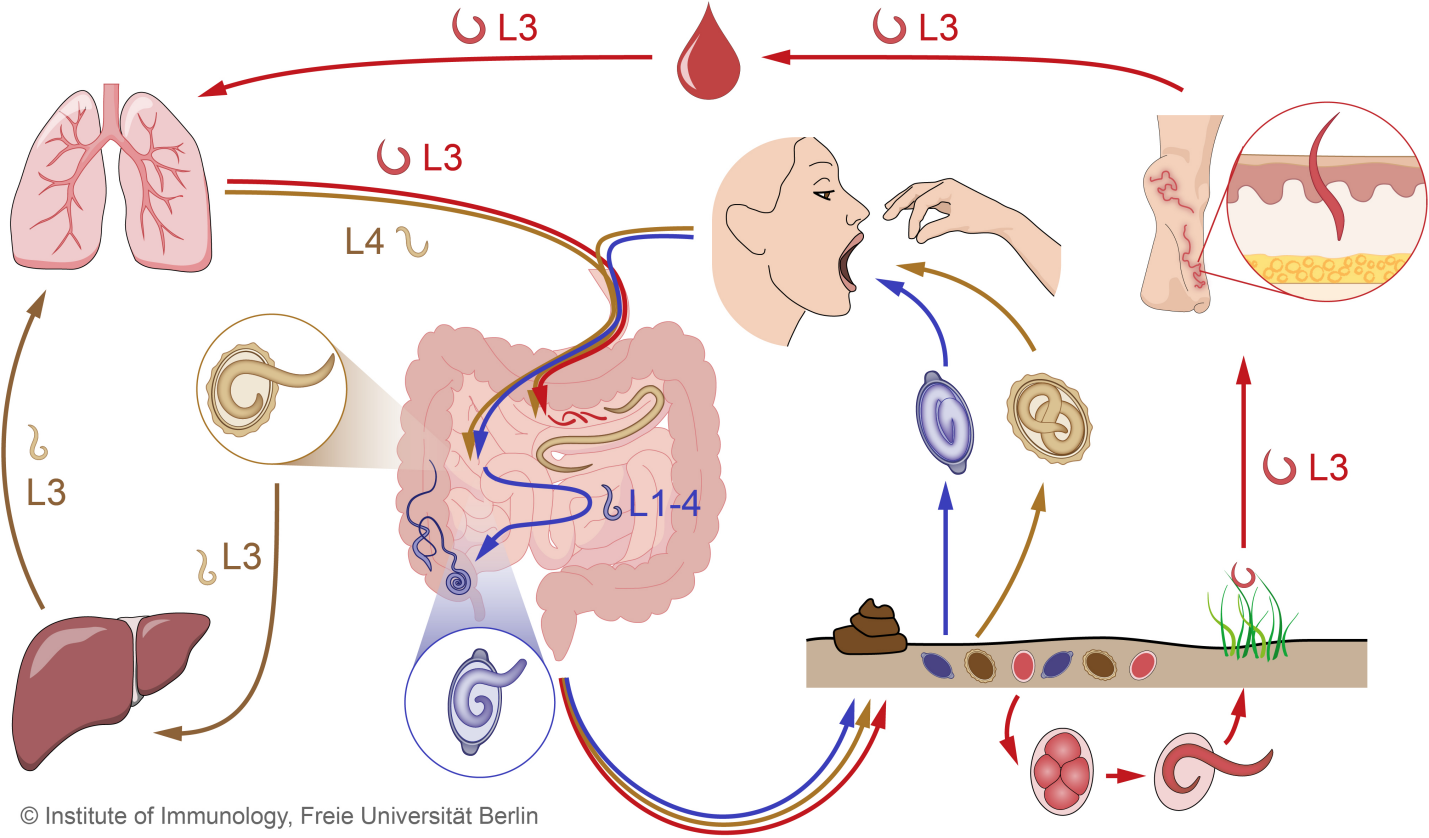
Combined STH life cycles for hookworms, *Trichuris trichiura* and *Ascaris lumbricoides*. Unembryonated eggs of all three STHs are excreted in the feces and develop into different larval stages over time in the soil. The infective eggs of *A. lumbricoides* (beige) and *T. trichiura* (purple), which contain the 3^rd^ and 1^st^ larval stages (L3, L1), respectively, enter the small intestine after oral ingestion, where the larvae hatch. In the case of hookworm eggs (red), the larvae hatch while still in the environment and develop into infective L3. When hookworm L3 come into contact with the human host, they penetrate the skin and are transported through the blood vessels to the heart and then to the lungs. Similar to hookworms, *Ascaris* larvae undergo extensive tissue migration. The L3 enter the proximal parts of the colon and migrate to the liver *via* the portal blood stream before reaching the lungs *via* systemic circulation. In the lungs, *Ascaris* L3 and hookworm L3 enter the alveoli, ascend the bronchial tree to the pharynx, and are swallowed. *Ascaris* L4 and hookworm L3 enter the small intestine, where they mature into adult worms. Adult *Ascaris* worms live exclusively in the lumen of the small intestine, while hookworms attach to the intestinal wall to feed on blood. *Trichuris* larvae mature intestinally without body migration and settle as adults in the colon, where they penetrate the mucosa with the anterior part. In the intestine, adult females of all three STHs produce eggs that are shed with the feces. STH, soil-transmitted helminth.

Humans are susceptible to more than 1,200 pathogens, including viruses, bacteria, helminths, protozoa, and fungi. Coinfection with multiple pathogens is common and can result in a range of interactions, either beneficial or antagonistic for the host. Soil-transmitted helminths (STHs) are potent immunomodulators, yet evidence on the effects of STH infections on the direction and extent of concurrent infections with other pathogens is inconsistent. STHs refer to intestinal worms that infect humans and animals alike, and are transmitted through contaminated soil (**Figure 1**). A large proportion of the world’s population is infected with one or more of these STHs: ~807-1,121 million people with roundworms (*Ascaris lumbricoides*), ~604-795 million with whipworms (*Trichuris trichiura*), and ~576-740 million with hookworms (*Ancylostoma duodenale* and *Necator americanus*) (Centers for Disease Control and Prevention, 2022).

STH infections occur primarily in areas with warm and humid climates where sanitation and hygiene are inadequate. STHs are considered neglected tropical diseases (NTDs), causing tremendous disability and suffering but can be controlled or eliminated. Coinfections of STHs with other pathogens (e.g. viruses, bacteria, protozoa, other helminths) represent an important clinical and epidemiological problem, especially in children suffering from high parasite burdens. In many cases, the pathogenic mechanisms of these coinfections are not fully understood, and the results obtained are inconclusive. The biology and current disease burden, pathogenesis, and efforts to combat STH were recently reviewed (Loukas et al., 2021). This review highlights the epidemiology of STH coinfections with a focus on Kenya, where the high disease burden was recently documented in nationwide surveys of the prevalence of STH and other infectious diseases. Human data will be complemented by studies in animal models to outline the various mechanisms by which helminths and associated immune responses interact with different microbial pathogens and other helminth species. Prevalence and mechanistic data concerning coinfections with schistosomes, a group of helminths not belonging to the STHs, were included where appropriate. The review concludes with a discussion on therapeutic interventions and future directions.

### 1.1 Epidemiology of STH and coinfections in Kenya

#### 1.1.1 Prevalence of STH infections

**Figure 2:**
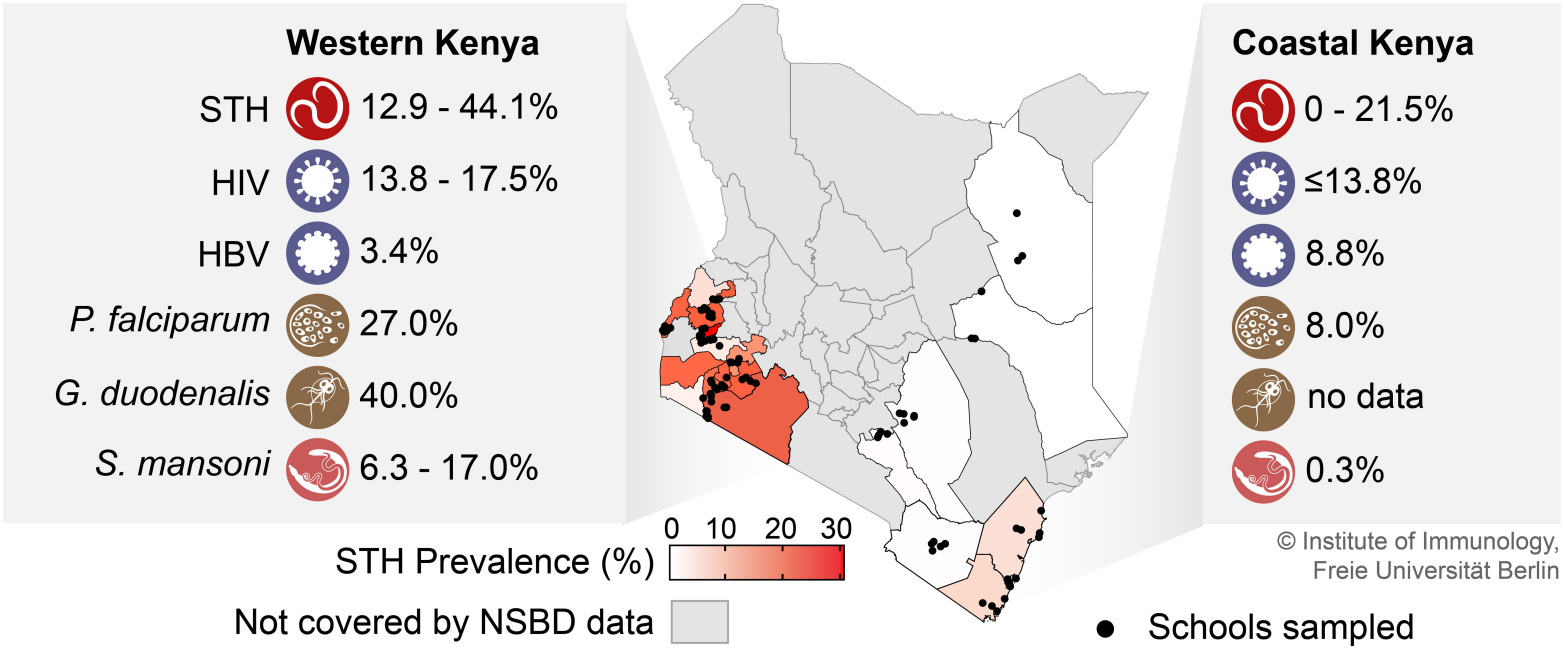
STH prevalence in Kenyan counties and co-occurring infections in areas of high STH prevalence (Western and Coastal Kenya). Prevalence data shown here for STH and *S. mansoni* infections were adapted from the 2018 survey for the National School-Based Deworming (NSBD) Program in Kenya (Okoyo et al., 2020) complemented with data from (Ngonjo et al., 2016; Halliday et al., 2019; Masaku et al., 2020; Araka et al., 2022; Kepha et al., 2023). The prevalence data for the co-occuring viral infections were taken from (Walson et al., 2010; Storey et al., 2017; Awili et al., 2020; Onyango et al., 2021; Wahome et al., 2022), and for the co-occurring protozoan infections from (Kepha et al., 2015; Pickering et al., 2019; Division of National Malaria Programme (DNMP) [Kenya] and ICF, 2021; Kamau et al., 2021). NSBD, National School-Based Deworming Program; STH, soil-transmitted helminth; HIV, human immunodeficiency virus; HBV, hepatitis B virus; *P. falciparum*, *Plasmodium falciparum*; *G. duodenalis*, *Giardia duodenalis*; *S. mansoni*, *Schistosoma mansoni*.

Low- and middle-income countries are particularly burdened by infectious diseases caused by pathogens such as helminths, viruses, bacteria, and protozoa (Bygbjerg, 2012). In Kenya, STH infections are common and most prevalent in western and coastal regions (**Figure 2**). In STH endemic areas in Kenya, multiple infections with different pathogens are very likely, although the prevalence of coinfections and their interaction are often poorly studied. The majority of published data on STH prevalence are from school-aged children, collected mainly through the National School-Based Deworming (NSBD) Program in Kenya. Notably, the STH non-endemic regions in Kenya are not covered by the NSBD Program due to the anticipated low prevalence and transmission rates (Pullan et al., 2011; Okoyo et al., 2016), hence there is a conspicuous lack of STH prevalence data for these regions from both the NSBD Program and other studies. The most recent NSBD survey reported an overall STH prevalence of 12.9% among children aged 4-15 years from six regions of Kenya in 2020 (Okoyo et al., 2020). In the same period, *A. lumbricoides* was the most prevalent STH species at 9.7%, while *T. trichiura* and hookworms accounted for 3.6% and 1.0%, respectively. Other individual studies in children of similar ages reported STH prevalences of up to 44.05% and 20.9% in Western and Coastal Kenya, respectively, with *A. lumbricoides* and hookworm infections predominating (Ngonjo et al., 2016; Halliday et al., 2019). Notably, STH prevalence in Coastal Kenya for 2020 was slightly higher among children aged 4-15 years using precision mapping (Kepha et al., 2023), highlighting the importance of the sampling method in assessing STH prevalence. Although preschool-aged children and adults may act as reservoirs for STH transmission, there are limited data on STH prevalence in these age groups in Kenya. The reported prevalence of STH among preschool children in Busia County, Western Kenya, was 17.0% in 2020 (Masaku et al., 2020). A recent study found an STH prevalence of 12.4% among pregnant women in Western Kenya (Araka et al., 2022). With the introduction of mass drug administration (MDA) programs in several helminth endemic countries, the prevalence of severe STH infections has gradually declined to moderate and mild infections (Mwandawiro et al., 2019). Nevertheless, the actual STH prevalence in Kenya may be slightly higher than reported because most STH prevalence studies use the Kato-Katz method for screening, which provides low sensitivity to low infection intensities (Okoyo et al., 2018).

#### 1.1.2 Prevalence of STH-virus coinfections

While there is extensive knowledge about the burden of helminth and viral infections, relatively little is known about the combinations of helminth and viral infections in Kenya. Here, due to the high burden of HIV/AIDS in STH endemic regions, most studies have focused on the prevalence of STH-HIV coinfection, while data on coinfection with other viruses, particularly enteric, respiratory and hepatotropic viruses, are largely lacking. HIV infection rates in Kenya are highly heterogeneous across the country, with prevalence in Kisumu, Western Kenya, at 17.5%, more than three times the national average (Onyango et al., 2021). Two studies of STH-virus coinfection in Kenya have reported a prevalence of 15.5% to 19.3% (Walson et al., 2010; Storey et al., 2017); however, there were regional differences in the prevalence of STH-HIV coinfection, ranging from 13.8% in the rural coastal and western regions to 44.7% in the urban regions of Nairobi (Walson et al., 2010). Regarding the most common STHs found in HIV-infected individuals, *A. lumbricoides* was the most common in the majority of studies, followed by hookworms and *T. trichiuria*. Worldwide, hepatitis A virus (HAV), hepatitis B virus (HBV), and hepatitis C virus (HCV) account for most cases of viral hepatitis, with hepatitis E virus (HEV) emerging and often being underdiagnosed (Shin et al., 2016; Kamar et al., 2017). In particular, the burden of HBV remains enormous in Kenya, with a recent meta-analysis finding an estimated overall prevalence of 7.8% (Makokha et al., 2023). In highly STH endemic regions, HBV prevalence ranged from 3.4% in Western Kenya (Awili et al., 2020) to 8.8% in Coastal Kenya (Wahome et al., 2022). However, despite the clustered regional occurrence, there have been no studies of STH coinfection with hepatotropic viruses in Kenya.

#### 1.1.3 Prevalence of STH-bacteria coinfections

The Global Burden of Disease Study found an estimated 7.7 million deaths, or 13.6% of all global deaths, were associated with bacterial pathogens in 2019 (Ikuta et al., 2022). Importantly, Kenya was listed as a high burden tuberculosis state (World Health Organization, 2016), with a reported prevalence of bacteriologically confirmed pulmonary TB of 558 per 100,000 population (Enos et al., 2018). Furthermore, different parts of Kenya have experienced multiple cholera outbreaks (Mutonga et al., 2013; Kiama et al., 2023), and many cases of infectious diarrhea were linked to bacterial enteropathogens, including diarrheagenic *Escherichia coli, Shigella, Salmonella*, and others (Shah et al., 2016). While there are a number of publications reporting the prevalence of STH in Kenya, few have examined the prevalence of bacteria in the same studies. A literature search on helminth-bacterial coinfections in Kenya did not reveal a single study. The closest study examined the effects of anthelminthic treatment on the gut microbiota. This study revealed that the presence of *A. lumbricoides* or *N. americanus* did not alter the overall microbiota composition compared to age-matched controls (Easton et al., 2019). Consequently, the prevalence and impact of STH-bacteria coinfections in this country remain unknown.

#### 1.1.4 Prevalence of STH-protozoa coinfections

Similar to STH, *Plasmodium falciparum* is also endemic in Western and Coastal Kenya. Here, the prevalence of malaria is 27.0% in the western region and 8% in the coastal region (Division of National Malaria Programme (DNMP) [Kenya] and ICF, 2021). The prevalence of STH-*P. falciparum* coinfection among school children in Bungoma in Western Kenya was at 14.3%, with the majority of malaria infections reported asymptomatic (Kepha et al., 2015). In addition to STH infections, Kisumu County, also in the western region of Kenya, reported a prevalence of 18% *Schistosoma mansoni*-*P. falciparum* coinfections among adults aged 18-35 years (Kamau et al., 2021). The prevalence of *Giardia* among children aged 2-5 years in the western region was at 40% (Pickering et al., 2019), whereas no data on *Giardia* prevalence are available for the coastal region of Kenya.

#### 1.1.5 Prevalence of STH coinfections with other helminths

Other helminths such as *S. mansoni* are also common in Western and Coastal Kenya with prevalence of 6.3% and 0.3%, respectively, although prevalence in western counties of Kenya can be as high as 17.0% depending on the distance from water bodies (Masaku et al., 2020; Okoyo et al., 2020). Despite the widespread geographic overlap of various STHs and other helminths in Kenya, most studies of STH prevalence in Kenya generally report the prevalence of infections with a single STH species. One study in Western Kenya found a coinfection prevalence of 4.7% among schoolchildren with hookworms and *A. lumbricoides* (Kepha et al., 2015). Studies in the elderly population show a relatively lower prevalence of STH coinfection with other helminths, likely due to the expected lower prevalence of STH in adults. One such study among women of childbearing age in Kwale County, Coastal Kenya, found a prevalence of *Schistosoma haematobium* and STH coinfection of 0.4% (Jeza et al., 2022).

### 1.2 Mechanisms of immunity to STHs

Gut-dwelling STHs modify the microenvironment of the gastrointestinal tract, including the epithelial cell layer and immune cells located in the underlying stroma, but also the surrounding microbiota (Zaph et al., 2014; Harris and Loke, 2018; Loke et al., 2022). In addition, certain STHs, such as *A. lumbricoides*, *A. duodenale*, and *N. americanus*, undergo an extraintestinal phase in which larvae migrate through different tissues before developing into sexually mature adult worms in the intestine (Vacca and Le Gros, 2022).

**Figure 3:**
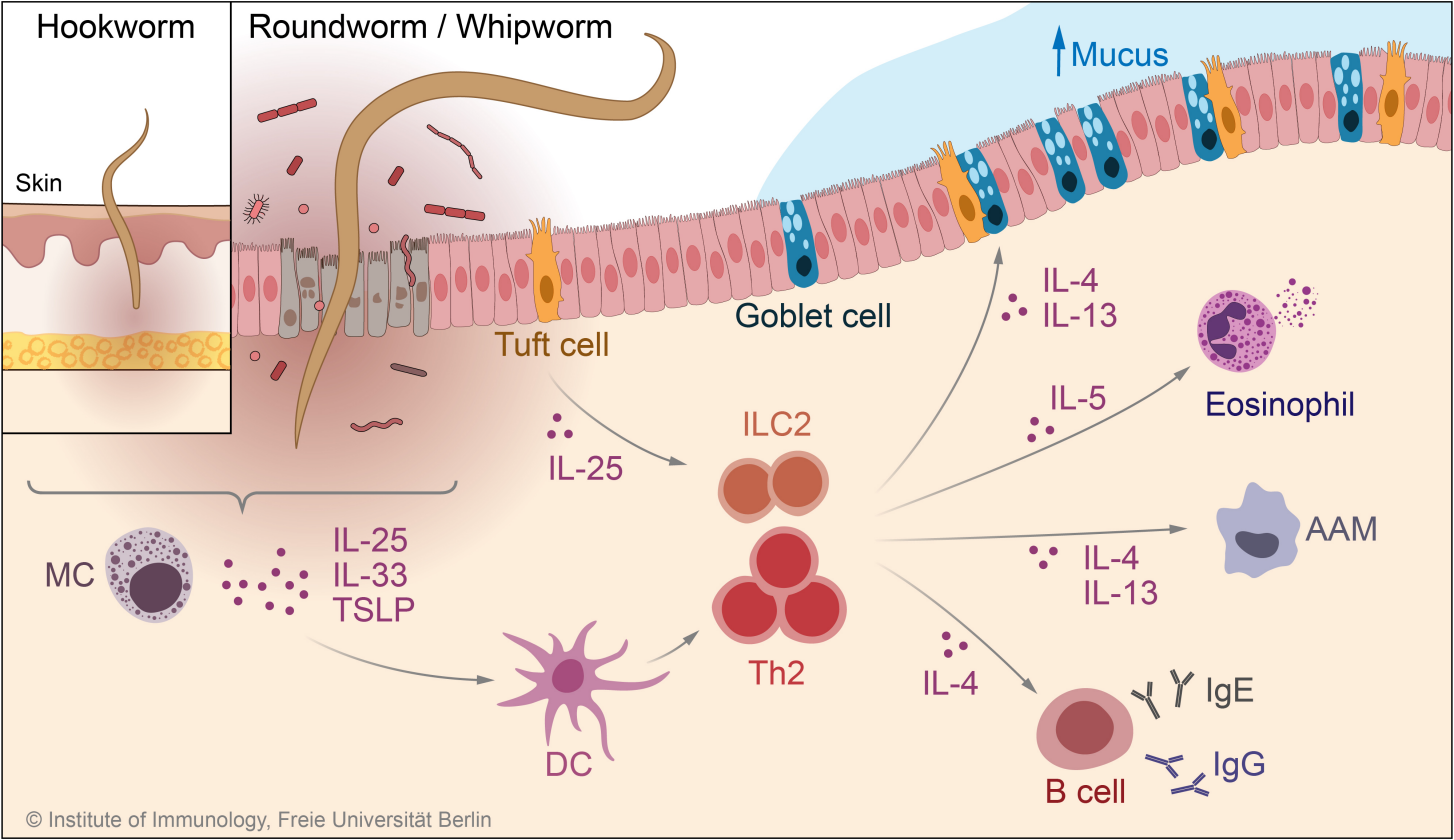
Immune mechanisms in response to infection with STHs. During anti-STH immunity, epithelial cell- and mast cell-derived cytokines such as TSLP and IL-33, together with tuft cell-derived IL-25, lead to activation of the type 2 response by ILC2 and Th2 cells. Upon activation, these cells produce the type 2 cytokines IL-4, IL-5, and IL-13, which in turn activate effector mechanisms involved in the defense against worm infection, such as goblet cell hyperplasia and, increased mucus production, eosinophilia, and IgE/IgG production by B cells. MC, mast cell; TSLP, thymic stromal lymphopoietin; DC, dendritic cell; AAM, alternatively activated macrophage; ILC2, type 2 innate lymphoid cell.

Following host invasion, worm-induced barrier damage leads to the release of alarmins (TSLP, IL-25, IL-33) from epithelial, mesenchymal, and innate cells (Hepworth et al., 2012; Peng et al., 2022). These early events drive the activation and proliferation of innate immune cells, such as mast cells, dendritic cells, and type 2 innate lymphoid cells (ILC2s), promoting the induction of an adaptive type 2 (Th2) immune response (Peng et al., 2022). In parallel, epithelial tuft cells have recently been shown to be important regulators of the host response to various infections in the gut (Strine and Wilen, 2022). Tuft cells respond to helminth infection by releasing IL-25, which drives the release of IL-5, IL-13, and IL-4 by activated ILC2s (Colombo and Grencis, 2020; Hendel et al., 2022). Subsequently, Th2 cells and ILC2s amplify IL-4 and IL-13 signaling to activate host protective responses at the epithelial barrier, including tuft cell differentiation, hyperplasia of mucin-producing goblet cells, and increased epithelial cell turnover (Colombo and Grencis, 2020). IL-4/-13 and IL-5 produced by Th2 cells also promote the expansion of alternatively activated macrophages (AAM) and the recruitment of eosinophils into affected tissues (Peng et al., 2022). In the gut, IL-4 and IL-13 increase smooth muscle hypercontractility, embodying the “weep-and-sweep” response that eliminates luminal parasites (Sorobetea et al., 2018). Furthermore, IL-4 derived from follicular T-helper cells contributes to effective anti-parasitic immunity in promoting IgG and IgE production by B cells (Zaini et al., 2021). Immune mechanisms directed to STH infections are summarized in **Figure 3**.

## 2 Affected host tissues and immunomodulation by STH coinfections

The type of antiparasitic immune response and tissue damage by migrating larval stages of STH are likely to be key elements in determining the outcome of concurrent viral, bacterial, protozoan or helminth infections. As an example, helminths and viruses elicit different innate and adaptive immune signaling pathways that may antagonize each other (Maizels and Gause, 2014; Desai et al., 2021; Midha et al., 2021). Furthermore, worms interfere with the host immune response through the release of immunomodulatory molecules, creating an environment beneficial to the parasite. Immunomodulation may act at the expense of antimicrobial immunity, but in some circumstances may also reduce the immunopathology caused by the microbe. However, simultaneous inflammatory processes driven by coinfections in the same organ system may also exacerbate tissue damage and thus directly affect tissue integrity. In this context, the timing of coinfection relative to the stage of the helminth life cycle, tissue tropism, interaction with the host microbiota and the extent of helminth-driven type 2/regulatory responses play key roles in determining the outcome of coinfection. Nevertheless, the frequency of these coinfections and associated comorbidities and the mechanisms of interactions in humans are poorly understood. Access to organ material is a limiting factor, and studies in animal models can provide valuable mechanistic information in this regard.

In the following sections, human-relevant STH coinfections with viruses, bacteria, protozoa, and other helminths are discussed according to the major organ systems affected, with a particular focus on the most important emerging and reemerging diseases occurring in Africa (Karagiannis-Voules et al., 2015; Fenollar and Mediannikov, 2018; Chauhan et al., 2020; Siwila et al., 2020; Afolabi et al., 2022; Nyaruaba et al., 2022). Where available, human studies are supplemented with data from animal models.

## 3 STH coinfections with viruses

In endemic areas, coinfections of helminths with certain viral diseases are common, causing different pathologies and associated immune responses depending on the helminth species, viral cell tropism and the timing of coinfection (Desai et al., 2021). Despite the frequent co-occurrence of STHs and viral infections, there is little information on their interactions when coinfected in humans.

### 3.1 STHs and enteric viruses

Worldwide, and thus also in helminth endemic areas, enteric viruses such as noroviruses and rotaviruses are the main cause of severe acute gastroenteritis in children under five years of age (Djeneba et al., 2007; Potgieter et al., 2023). Reinfection is common, with virus-specific IgA correlating with protection and increasing over time, whereas IFN-γ-dominated specific T cell responses are transient (Malm et al., 2019). However, the effects of STH coinfection with intestinal viruses in humans are not well understood. In mice, acute enteric helminth infections with *Heligmosomoides polygyrus* and *Trichinella spiralis* resulted in impaired virus-specific CD8+ T cell responses (Osborne et al., 2014). This defect was STAT6-dependent, associated with the nematode-triggered alternative activation of macrophages, and was partially restored by neutralization of Ym-1, a product released by AAM (Osborne et al., 2014). Another study reported the increased enteric replication of flaviviruses in *H. polygyrus*-infected mice which was associated with a tuft cell-IL-4 receptor signaling axis leading to dampening of antiviral adaptive immunity (Desai et al., 2021). Associated with goblet cell hyperplasia during murine *H. polygyrus* coinfection, murine astrovirus replication and shedding was also enhanced (Ingle et al., 2021).

### 3.2 STHs and hepatotropic viruses

HAV, HBV, and HCV are responsible for most cases of viral hepatitis, with HEV emerging and often being underdiagnosed (Shin et al., 2016; Kamar et al., 2017). Thus, a correlation between human infections with *A. lumbricoides* and HBV has been demonstrated (Xiao et al., 2015), but the effects on correlated immune responses and liver pathology are unknown. To date, studies addressing the potential impact of helminth infections on the controls of hepatotropic viruses focused on patients coinfected with schistosomes. Interestingly, HEV seroprevalence was very high among Egyptian workers and a Brazilian cohort infected with schistosomes (el-Esnawy and Al-Herrawy, 2000; Passos-Castilho et al., 2016). Here, previous HEV infection was associated with a higher frequency of liver enzyme abnormalities (Passos-Castilho et al., 2016), indicating long-lasting effects of HEV. Similarly, concurrent infections with HBV and HCV in trematode endemic areas are often observed (Abruzzi et al., 2016; Gao et al., 2020). Related to increased Th2 cytokines, patients with concurrent helminth and HBV or HCV infection showed increased viral replication and liver fibrosis (Abruzzi et al., 2016; Li et al., 2016; Dong et al., 2022). Hence, chronic inflammatory processes associated with hepatic viral replication next to Th2-mediated fibrotic responses to Schistosome eggs may result in more extensive liver damage caused mainly by immune-mediated mechanisms (Shin et al., 2016). Furthermore, the modulation of early intrahepatic antiviral immune pathways (e.g. type I IFN) in murine helminth-virus coinfection was shown to be associated with increased viral replication in the liver (Edwards et al., 2005). Whether recurrent tissue damage induced by migrating *Ascaris* larvae has a similar effect remains to be investigated. Furthermore, it is unclear whether tissue damage induced by blood-feeding hookworm species may affect the immune response to hepatic viruses. *A. lumbricoides*, *A. duodenale* and *N. americanus*, have an extraintestinal phase in which the larvae migrate through various tissues before developing into sexually mature adult worms in the gut. The liver, as an organ that preferentially induces tolerance to immunity, is passed by *Ascaris* during its life cycle before the larvae reach the lungs with liver larvae attrition occurring under certain circumstances (Midha et al., 2021). This function can be exploited by parasites to evade host immunity. Thus, hepatotropic viruses that exploit similar strategies preferentially infect hepatocytes.

### 3.3 STHs and respiratory viruses

Tissue-invasive STHs that migrate through the lungs evoke inflammatory changes and tissue damage that can negatively impact concurrent respiratory viral infections or even pave the way for secondary infections. Respiratory syncytial virus (RSV) is the most common cause of acute respiratory diseases in children worldwide (Gatt et al., 2023). An imbalance in the Th1/Th2 cytokine immune response has been associated with the RSV pathogenesis of bronchiolitis (Pinto et al., 2006). Thus, Th1/Th2 ratios, helminth-specific-IgE and TLR4-gene-polymorphisms are related to infant RSV disease severity in parasite-endemic areas (Caballero et al., 2015; Buendía et al., 2022). Research is also underway to determine how coinfection with STHs can influence inflammatory immune activity in SARS-CoV-2 infection and thus disease severity, but also the efficacy of vaccination against SARS-CoV-2 (Adjobimey et al., 2022; Akelew et al., 2022). Interestingly, the prevalence and mortality due to COVID-19 have remained moderate on the African continent (Cepon-Robins and Gildner, 2020). However, *Ascaris* antigens affected immune reactivity to SARS-CoV-2 peptides by reducing virus-reactive Th cells and type 1 cytokines in COVID-19 patients, suggesting a negative impact of concurrent helminth infestation on antiviral immunity (Adjobimey et al., 2022). In line, *Ascaris*-infected pigs were shown to be more susceptible to respiratory virus infection (Oba et al., 2023). Indicating a bidirectional interaction, pulmonary *Ascaris* and vaccinia virus coinfection in mice weakened virus-specific immunity and not only exacerbated virus-associated pathology, but also reduced lung larval loads (Gazzinelli-Guimarães et al., 2017). However, in addition to the detrimental effects in STH coinfections, strictly enteric helminth infection with *H. polygyrus* protected mice from severe pulmonary RSV infection by microbiota-dependent type I interferon production, with Th2 responses dispensable (McFarlane et al., 2017).

### 3.4 STHs and viruses with immune cell tropism

Herpesviruses have a marked tropism for immune cells and latently infected cells remain for life with virus reactivation occurring under certain circumstances (Lan and Luo, 2017). Because of their high prevalence, many people are coinfected with herpesviruses and STHs, and although both pathogens modulate the host’s immunity (Peacock et al., 2003; Barton et al., 2007; Lan and Luo, 2017), their interactions in humans remain largely unexplored. The local cytokine environment during acute helminth infection alters latent herpesvirus infection, whereby IL-4 reactivated human Kaposi’s sarcoma-associated herpesvirus from latency in cultured human cells (Reese et al., 2014). The extent of immunity to helminth infection may be relevant here, as no reactivation of latent Epstein-Barr virus infection was observed in multiple sclerosis patients who underwent low-dose therapeutic oral hookworm administration (Maple et al., 2020). A “two-signal” model for viral reactivation was postulated, as exogenous IL-4 and IFN-γ-blockade reactivated latent murine gammaherpesvirus-68 (MHV68) infection *in vivo* (Reese et al., 2014), which required macrophage IL-4-receptor-expression. Accordingly, *Nippostrongylus brasiliensis* infected mice developed type 2 immune-driven enhanced pathology upon genital herpesvirus infection (Chetty et al., 2021), a finding also translatable to humans, since hookworm-infected women in an African cohort were at high risk for viral infections in the genital tract (Holali Ameyapoh et al., 2021). However, IL-4 also has beneficial effects on IFN-γ-dependent antiviral effector responses, as helminth-mediated (*N. brasiliensis*, *H. polygyrus*) conditioning of virtual memory CD8+ T cells (TVMs) enhanced control of murine gammaherpesvirus 4 (MHV-4) infection (Hussain et al., 2023). Of note, this helminth-mediated increase in TVMs is transient and absent in aged mice (Hussain et al., 2023) which may be particularly relevant to herpesvirus reactivation in elderly worm-infected patients.

Human retroviruses such as HIV cause damage to lymphoid and mucosal tissues, leading to progressive immunodeficiency (Heron and Elahi, 2017). Sub-Saharan Africa is burdened by HIV/AIDS and helminthiasis, and there is considerable overlap between these infections, with poor sanitation and socioeconomic status influencing the occurrence of HIV and STH coinfections (Mpaka-Mbatha et al., 2023). Compared to HIV-infected patients in Africa, adults coinfected with HIV and intestinal helminths (e.g. hookworms) have been found to have increased susceptibility to micronutrient deficiencies and anaemia, as well as higher viral loads and lower CD4+ T cell counts (Mkhize-Kwitshana et al., 2011; Morawski et al., 2017; Amoo et al., 2018; Wondmieneh et al., 2020). According to two studies in South Africa, HIV- and *A. lumbricoides*-coinfected individuals with high *Ascaris*-specific-IgE levels and eosinophilia displayed the highest viral loads and reduced levels of type 1 cytokines, indicating a suppressive effect of helminth-mediated immunity on HIV control (Mkhize-Kwitshana et al., 2011; Mkhize-Kwitshana et al., 2014). However, findings are conflicting, as serum cytokine profiling of HIV- and STH-coinfected pregnant women in Nigeria revealed increased IFN-γ and IL-17 levels (Rabiu et al., 2022). In line, Peruvian patients coinfected with another retrovirus, HTLV-1, and *Strongyloides stercoralis* have higher parasite burdens and lower type 2 immune responses than patients with strongyloidiasis alone (Montes et al., 2009).

**Figure 4:**
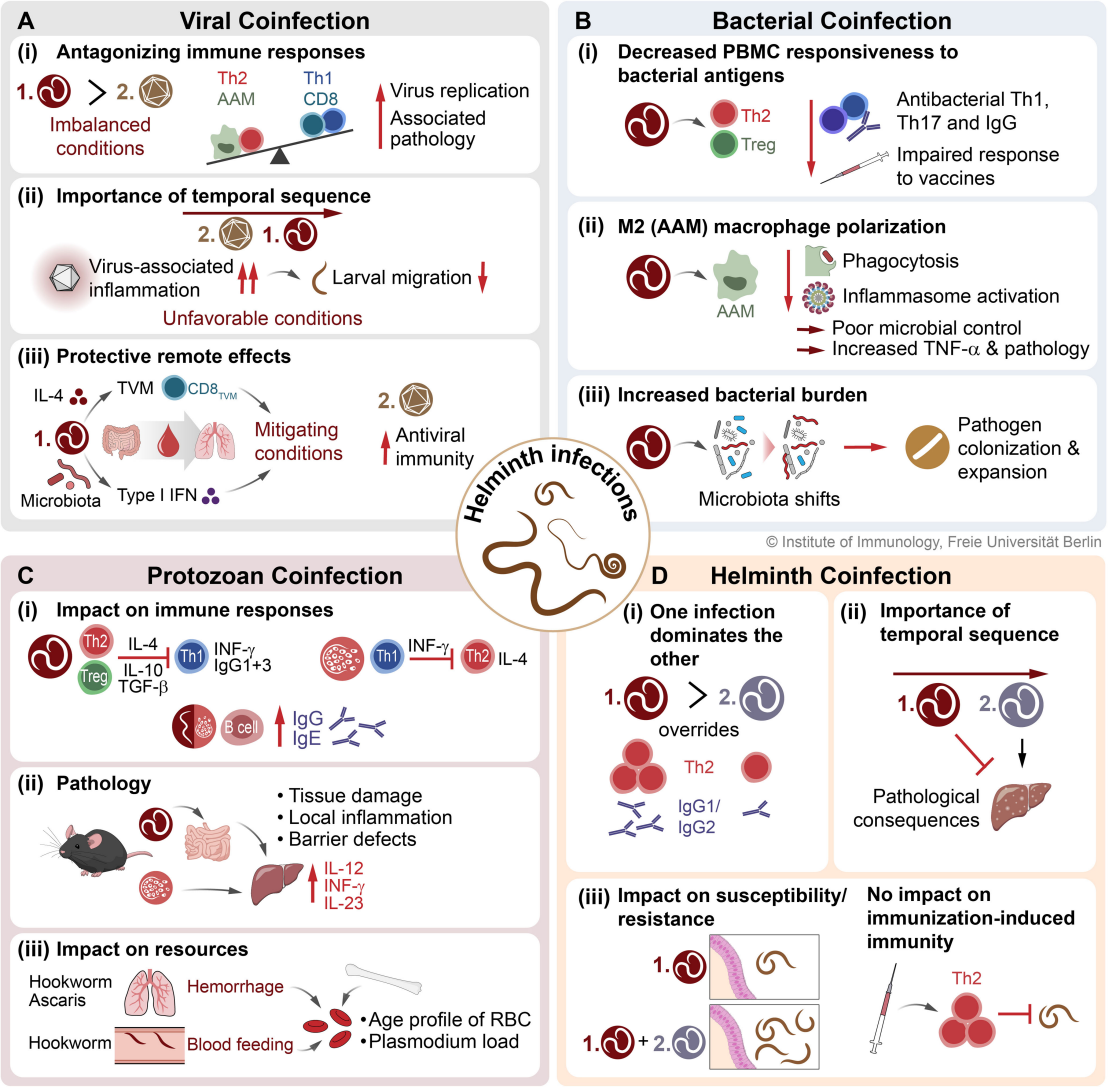
The consequences of STH coinfections on host immunity and infection outcome. Here, the interactions and associated effects on immune responses and pathologies of STH coinfections with **(A)** viruses, **(B)** bacteria, **(C)** protozoa (exemplary for *Plasmodium*), and **(D)** other helminths are presented. STH, soil-transmitted helminth; TVM, virtual memory CD8+ T cell; AAM, alternatively activated macrophage; CAM, classically activated macrophage; Th1, type 1 T helper cell; Th2, type 2 T helper cell; Treg, regulatory T cell; RBC, red blood cell.

In conclusion, STH coinfections with viruses can have profound effects on the outcome of each infection through the following interactions (**Figure 4A**): i.) STHs facilitate viral replication by remodeling tissue morphology and counteracting classical IFN-γ/TNF-mediated type 1 inflammatory pathways due to initiated type 2 immune circuits; ii.) Depending on the timing and extent of virus-induced inflammation, an unfavorable environment for migrating STH larvae is created, which may compromise the development of patent worm infection; iii.) STHs may also exert direct type 2 cytokine-mediated and indirect microbiota-dependent remote effects in non-infested organs, resulting in enhanced antiviral immunity that protects against virus-related pathology. Thus, infections with STH can alter host immunity and tissue integrity both directly in parasitized organs and indirectly in uninfected organs, and therefore have systemic effects that are highly complex and appear to depend largely on the context and biology of viral coinfection in a bidirectional manner.

## 4 STH coinfections with bacteria

Interactions between helminths and bacteria can occur at the site of infection in the intestine, in sites impacted by helminth tissue migration such as the lungs, or indirectly *via* the microbiota. STHs migrating through and residing in the intestine are surrounded by microbes and numerous studies have documented alterations to the host microbiota composition during helminth infections as well as direct interactions between STHs and microbes reviewed in (Midha et al., 2017; Midha et al., 2021). Here we consider data concerning enteric pathogens such as *Salmonella* spp. which may also become systemic in the case of typhoid fever, and respiratory pathogens such as *Mycobacterium tuberculosis.*

### 4.1 STHs and enteric bacteria

As with enteric viruses, coinfections with STHs and enteric bacteria, some of which share the fecal-oral route of transmission, are also widespread. Between human and animal studies of STH-bacterial coinfections as well as data from other helminths such as vector-borne filarial nematodes and trematodes such as *Schistosoma*, there is considerable evidence for helminth immunomodulation leading to increased bacterial burdens and pathology (Rocha et al., 1971; Gendrel et al., 1994; Bobat et al., 2014; Reynolds et al., 2017). The outcomes of coinfection can also be influenced by the pathogens in question as well as by timing. Mice coinfected with *S. mansoni* and *Listeria monocytogenes* displayed increased resistance to *Listeria* infection early in *Schistosoma* infection, and decreased resistance to bacterial infection later in the course of *Schistosoma* infection (Collins et al., 1972).

Infection with *A. lumbricoides* is negatively associated with IFN-γ and IL-7 production in response to LPS as demonstrated in Tsimane hunter-horticulturalists (Schneider-Crease et al., 2021). This negative association is also associated with impaired responses to bacterial vaccines. PBMCs from teenage Ecuadorian patients infected with *A. lumbricoides* had impaired cholera toxin B-specific IL-2 and IFN-γ responses following vaccination with CVD 103-HgR, a live attenuated *Vibrio cholerae* strain used for cholera vaccination (Cooper et al., 2001). Interestingly, anthelmintic treatment of the same ascariasis patients enhanced the vibriocidal antibody response (Cooper et al., 2000). In pigs, *A. suum* infection negatively affects the protective response of *Mycoplasma hypopneumoniae* vaccination, including impaired seroconversion and antibody titres, while increasing lung pathology (Steenhard et al., 2009).

Clinical observations indicate that coinfection with *Schistosoma* and *Salmonella* is quite common; helminth-infected Gabonese children and rural Congolese villagerss were more often found to carry *Salmonella* (Gendrel et al., 1994; Mbuyi-Kalonji et al., 2020) and schistosomiasis patients were found to suffer from prolonged bacteremia and salmonellosis (Rocha et al., 1971), a situation which can be improved by anthelmintic treatment (Hathout et al., 1966; Gendrel et al., 1984). These observations are well supported by coinfection studies in mice where *Schistosoma-Salmonella* coinfected mice have higher bacterial burdens and increased mortality compared to mice infected only with *Salmonella* (Njunda and Oyerinde, 1996). Importantly, similar outcomes are observed in STH-*Salmonella* coinfection, including with *N. brasiliensis* (Bobat et al., 2014) and *H. polygyrus* (Reynolds et al., 2017) wherein helminth-impaired host resistance to *Salmonella* is restored by deworming (Brosschot et al., 2021).

Protective responses against *Salmonella* are typically initiated by bacterial recognition *via* patterns-associated molecular patterns such as lipopolysaccharide (LPS), type-3 secretion system proteins, flagella, fimbriae, and bacterial DNA combined with endogenous danger-associated molecular patterns, leading to neutrophil and macrophage recruitment and pro-inflammatory cytokine secretion, including IL-1β, IL-6, IL-17, TNF-α, and IFN-γ (de Jong et al., 2012). Innate antibacterial responses by neutrophils and macrophages can be influenced by helminth infection. Su and colleagues demonstrated that *H. polygyrus* can worsen intestinal inflammation during *S.* Typhimurium infection by compromising neutrophil recruitment and control of bacterial replication (Su et al., 2014). Furthermore, helminth antigens trigger IL-4 and IL-13 release by basophils leading to alternative activation of macrophages, impaired IFN-γ and IL-17 production, and thus impaired anti-Salmonella protective Th1/Th17 responses (Knuhr et al., 2018; Schramm et al., 2018). Similar observations have been made in mice coinfected with *H. polygyrus* and *Citrobacter rodentium*, a gram-negative model to investigate enteropathogenic and enterohaemorrhagic *E. coli* (EPEC and EHEC, respectively) infections and colitis (Collins et al., 2014), where coinfected mice display considerable morbidity and mortality (Chen et al., 2005) and higher bacterial loads compared to single-infected mice (Weng et al., 2007). The Th2-driven alternative activation of macrophages resulted in downregulated protective IFN-γ and corresponding impaired bacterial killing alongside worsened colitis mediated by enhanced TNF-α responses (Chen et al., 2005; Weng et al., 2007). *H. polygyrus* infection can also promote *S.* Typhimurium infection independently of a modulated Th2 response. Reynolds and colleagues demonstrated the host intestinal metabolome was altered by *H. polygyrus* to enhance the expression of *Salmonella* pathogenicity island 1 genes, leading to increased bacterial invasion (Reynolds et al., 2017).

Interestingly, bacterial infection can also impact parasite burdens. Mice coinfected with *S. japonicum* and *S.* Typhimurium had reduced adult worm burdens and reduced mortality compared to *S. japonicum* infection alone (Zhu et al., 2017). In these mice, *Schistosoma* infection was associated with increased serum IFN-γ and IL-4 levels compared to uninfected controls while coinfection further increased IFN-γ, but reduced IL-4 to uninfected levels (Zhu et al., 2017). Another important consideration is the order of infection. In the case of *N. brasiliensis*-*S.* Typhimurium coinfection, an established helminth infection impairs control of subsequent bacterial infection while simultaneous coinfection impaired protective immunity to both pathogens despite robust antibody responses (Bobat et al., 2014). Interestingly, *Salmonella* infection restrained the regulatory response induced by *N. brasiliensis* as reflected by reduced IL-10 secretion by splenocytes (Bobat et al., 2014).

### 4.2 STHs and respiratory bacteria

The lung is a major site of immunomodulation with an impact on coinfecting bacteria. Clinical outcomes of helminth-respiratory bacterial coinfection are mixed though there is ample evidence to suggest that as with intestinal infections, helminth immunomodulation alters responses to respiratory pathogens as well. Higher prevalence of helminth infections have been reported in Ethiopian and Brazilian patients with active tuberculosis (TB) compared to matched controls (Tristão-Sá et al., 2002; Elias et al., 2006) as well as eosinophilia, a prominent feature of helminth infections (Elliott et al., 2003). Urban Indian adults coinfected with *Strongyloides stercoralis* and *M. tuberculosis* have higher circulating levels of matrix metalloproteinases and tissue inhibitors of metalloproteinases compared to *M. tuberculosis* single infection, indicating that coinfection could result in greater degradation of the basement membrane thereby creating an immune privileged site for the growth of *M. tuberculosis* (Kathamuthu et al., 2021). In one study, STHs, namely *Ascaris* and *Trichuris*, were identified as a risk factor for increased pneumococcal carriage density in Ecuadorian children (Law et al., 2021) In the same report, *T. muris*-infected mice coinfected with *Streptococcus pneumoniae* had increased pneumococcal carriage density in the nasopharynx and enhanced bacterial dissemination into the lungs (Law et al., 2021). Another study found coinfection with *H. polygyrus* and *Bordatella bronchiseptica* led to higher bacterial loads in the lungs and higher mortality (Lass et al., 2013). Similarly, mice coinfected with *A. suum* (at day 8 post-infection during *A. suum’*s lung migration) and *Pasteurella multocida* developed more severe pneumonia and septicemia than did mice infected with bacteria alone (Tjørnehøj et al., 1992), suggesting that while STHs are actively migrating through the lungs, the host may be at an increased risk for bacterial coinfection.

As with *Salmonella*, resistance to mycobacterial infection is initiated with inflammatory responses by phagocytes triggered by pattern recognition receptors and control is mediated by Th1 and Th17 cells while the progression of tuberculosis is associated with the regulatory type-2 cytokines IL-4 and IL-10 (Babu and Nutman, 2016; Cadmus et al., 2020; Bustamante-Rengifo et al., 2021; Bhengu et al., 2022). Numerous studies have documented helminth-impaired reactivity to tuberculin antigens and compromised tuberculosis diagnostics (Babu and Nutman, 2016). Specifically, helminth infection appears to dampen the efficacy of Bacille Calmette-Guerin (BCG) vaccination, an attenuated strain of *Mycobacterium bovis*, and helminth infection can reduce mycobacterial-induced IFN-γ production in Bangladeshi children and pregnant Ethiopian mothers (Cadmus et al., 2020; Bhengu et al., 2022). *S. stercoralis*-*M. tuberculosis* coinfections in Indian adults are associated with lower circulating levels of inflammatory cytokines, including IFN-γ, TNF-α, IL-17A, and IL-17F alongside increased type 2 cytokines IL-4, IL-5, and IL-13 (George et al., 2015). Similarly, Indian TB patients coinfected with hookworms had fewer *Mycobacterium*-specific Th1 and Th17 cells and associated circulating cytokines (TNF-α, IFN-γ, IL-2, IL-17A) but increased Th2 cells and associated cytokines (IL-4, IL-13) as well as increased frequencies of regulatory T cells compared to patients infected with *M. tuberculosis* alone (George et al., 2013).

In conclusion, helminth coinfections can modulate antibacterial immune responses against bacterial pathogens in different organs. A general picture emerges whereby a helminth-induced regulatory type 2 response dampens cellular responses to bacteria and their antigens, polarizes macrophages away from an antibacterial proinflammatory state, and alters antibody responses against bacteria, thus establishing an environment better suited to the proliferation of bacterial pathogens (**Figure 4B**).

## 5 STH coinfections with protozoa

### 5.1 STH and malaria infection

Malaria as well as STH infections remain highly endemic in sub-Saharan Africa. A number of studies investigated the potential impact of STH infection on malaria prevalence, infection intensity and the associated immunopathology in humans as well as in experimental animal models. Several studies reported that infection with *A. lumbricoides*, *T. trichiura*, and hookworms were associated with an increased risk for the development of clinical, non-severe malaria (Nacher et al., 2002; Spiegel et al., 2003; Degarege et al., 2012; Zeukeng et al., 2014; Babamale et al., 2018), which was further increased with the number of coinfecting intestinal helminth species (Nacher et al., 2002; Degarege et al., 2012). Similarly, studies performed in murine models reported higher peak parasitemia in mice coinfected with *H. polygyrus* or *N. brasiliensis* (Su et al., 2005; Noland et al., 2008; Helmby, 2009; Hoeve et al., 2009; Segura et al., 2009; Tetsutani et al., 2009), and, in some cases, accelerated mortality associated with uncontrolled liver pathology (Helmby, 2009; Craig and Scott, 2017). By contrast, studies in humans found a protective effect of *Ascaris* and hookworm infection against the development of severe malaria as well as malaria-related acute renal failure (Nacher et al., 2000; Nacher et al., 2001). Timing is important for the outcome of GI nematode/*Plasmodium* coinfection in mice. Nematode infection one to two weeks prior to infection with *P. chabaudi* had limited impact on the course of *Plasmodium* infection, whereas simultaneous coinfection led to a dramatic rise in mortality, associated with highly increased liver damage. The latter was dependent on the elevated expression of IL-12, IFN-γ and IL-23 in coinfected mice (Helmby, 2009). Increased mortality coincided with larval-driven intestinal barrier damage, suggesting that danger signals reaching the liver *via* the portal system promoted overt hepatic inflammation when mice were simultaneously coinfected with *Plasmodium* parasites (Helmby, 2009).

Several studies reported impaired IFN-γ responses in coinfected mice, arguing for a detrimental effect of type 2 responses driven by nematode coinfection (Su et al., 2005; Noland et al., 2008; Tetsutani et al., 2009). However, peak parasitemia was also increased in coinfected STAT-6^-/-^ mice incapable of inducing a Th2 response to *H. polygyrus* (Segura et al., 2009). Importantly, a placebo-controlled deworming trial in Indonesia reported increased immune responsiveness after anthelmintic treatment (Wammes et al., 2016). Regular deworming resulted in lower expression of the suppressive molecule CTLA-4 by T cells, coinciding with significantly increased *Plasmodium*-specific TNF-α/IFN-γ and elevated *Ascaris*-specific IL-2 production (Wammes et al., 2016).

While some studies determined a higher risk for anemia and low body weight in STH coinfected patients (Degarege et al., 2010; Zeukeng et al., 2014), others found no effect on *P. falciparum*-induced anemia (Abanyie et al., 2013; Babamale et al., 2018). Investigating experimental infections in mice, Griffith and colleagues reported suppressed immune responses, yet smaller *Plasmodium* populations in mice coinfected with *N. brasiliensis* (Griffiths et al., 2015). In this model, target cell availability exerted a stronger effect on the dynamics of malaria replication than suppression of IFN-γ and IgG2a responses, potentially linked to altered mean age and population size of red blood cells in mice afflicted with hemorrhage induced during migration of nematode larvae through the lung. Considering resource-mediated next to immune-mediated mechanisms may hence be essential in understanding the dynamics of helminth-*Plasmodium* coinfections (Griffiths et al., 2015).

Th1 responses associated with the production of cytophilic IgG antibodies (IgG1, IgG3) are important for the clearance of *Plasmodium* blood-stage infection (Torre et al., 2002; Courtin et al., 2009). By contrast, helminth infections predominantly cause the production of IgG4 and IgE in a Th2-dependent manner (Harris and Gause, 2011). Accordingly, several studies reported a negative effect of concurrent helminth infection on the humoral response to malaria, seen in the reduction of *P. falciparum*-specific IgG (Ateba-Ngoa et al., 2016), IgG1 and IgG3, and a rise in IgG4 (Roussilhon et al., 2010; Courtin et al., 2011). On the other hand, two studies found higher IgG1, IgG2 and IgG3 responses to *P. falciparum* antigens in schistosome coinfected individuals (Diallo et al., 2010; Tokplonou et al., 2020). Similarly, Amoani and colleagues reported a positive effect of hookworm coinfection on the levels of IgG3 directed against the malaria vaccine candidate GMZ2 at baseline and a decline in the antibody level after albendazole treatment (Amoani et al., 2021). Another recent study surveyed antibody responses directed against a broad array of *Plasmodium* as well as STH antigens in Mozambican children and found consistently higher IgG levels against *Plasmodium* and nematode antigens as well as higher total IgE levels in the coinfected group (Santano et al., 2021). In trend, the study confirmed earlier reports of higher *Plasmodium* density in coinfected children and found significantly higher *Trichuris* worm burdens in malaria-positive subjects. Finally, socioeconomic score and improved sanitary conditions clearly impacted overall IgE levels and IgG levels against some *Plasmodium* and helminth antigens (Santano et al., 2021). Hence, synergies may exist between the Th2-related support of antibody production in helminth infection and humoral responses directed against *Plasmodium* parasites. However, as the risk of being infected and *Plasmodium* density tend to increase with helminth coinfection, it seems that such synergistic effects reflect the increased exposure rather than protection of coinfected patients. Although the gut microbiota may play a role in *Plasmodium* infections and contribute to the complexity of coinfection with STH (Easton et al., 2020), further studies are needed to specify this interaction.

In conclusion, there are at least three potential interactions between helminths and *Plasmodium* coinfection (**Figure 4C**): i) By the induction of Th2 responses, STH coinfection may interfere with Th1/IFN-γ driven effector/cytophilic IgG1/IgG3 responses required for the control of *Plasmodium* infection. Conversely, a strong Th1-bias may hamper effective type-2 driven control of STH infections. However, coinfection was also reported to be associated with enhanced parasite-specific IgG and IgE responses compared to single infections; ii) Some animal models suggest that simultaneous tissue damage driven by malaria parasites and helminths may result in an adverse inflammatory response, worsening disease outcome. However, STH infection rather seems to protect from severe malaria in humans; iii) Coinfection with *Ascaris*/hookworms is associated with hemorrhage in the liver during lung stage infection. In addition, adult hookworms feed on host blood. This may alter the age profile of erythrocytes and thereby limit resources for *Plasmodium* species favoring matured blood cells for infection.

### 5.2 STH and giardia infection

*Giardia duodenalis* colonizes the small intestinal tract where the trophozoite stage attaches to epithelial cells. The infection is often asymptomatic, but may also be associated with diarrheal disease, leading to malabsorption and, under chronic exposure, stunted growth (Klotz and Aebischer, 2015; Fekete et al., 2021). Experimental *Giardia* infections are controlled by Th17 and IgA responses and elevated Th17 activity was also linked with protective immunity against human giardiasis (Dann et al., 2015; Saghaug et al., 2016; Paerewijck et al., 2019). Despite the fact that *Giardia* shares the small intestinal habitat with *Ascaris*, hookworms and threadworms, relatively few studies tested for potential interactions between enteric nematode and *Giardia* infections. Furthermore, although the prevalence of *Giardia* infection is often high in areas where STH and/or schistosome infections are highly endemic (Fofana et al., 2019; Geus et al., 2019; Archer et al., 2020), data on potential interference of immune responses directed against the two types of parasites are largely lacking.

A survey of *Plasmodium*, *Ascaris* and *Giardia* infection in Rwandan schoolchildren showed that parasitic coinfections were common, but the clinical picture was mostly associated with *Plasmodium* infection and not modified by *Ascaris* or *Giardia* coinfection. However, malnutrition was more pronounced in children coinfected with *Ascaris* and *Giardia* compared to individuals with single infections (Geus et al., 2019). Similarly, a study in subtropical Argentina observed an association between stunting in older children and the presence of enteric parasites, multi-parasitism and giardiasis (Rivero et al., 2018). A long-term survey of over 80 villages in Bolivia revealed an antagonistic relationship between STH and *Giardia* infection (Blackwell et al., 2013). Both hookworm and *Ascaris* infection were negatively associated with *Giardia* infection in a cross-sectional as well as in a longitudinal survey. However, anthelminthic treatment only resulted in a marginal rise of the risk of being infected with *Giardia* on the next visits (Blackwell et al., 2013). Concerning modifications of the immune profile, one study reported reduced IL-2, IL-12 and TNF-α serum levels in 3-year old children coinfected with *Ascaris* and *Giardia* compared with *Giardia* mono-infected individuals, while Th2 cytokine levels were not different from the controls in any of the groups (Weatherhead et al., 2017). Finally, high *Giardia*-specific IgG and IgE levels were seen in a cohort affected by light, but not moderate *Ascaris* infection and high IL-10 levels in individuals with moderate *Ascaris* infection associated with poor inflammatory cytokine responses against *Giardia* antigens (Hagel et al., 2011).

Hence, evidence of the detrimental effects of STH on *Giardia* infection and the associated sequelae exists. However, more work in experimental models is needed addressing immunological cross-regulation, competition for resources and potential changes in barrier functions, mucus composition and gut microbial communities in the context of STH-*Giardia* coinfections.

## 6 STH coinfections with other helminths

Coinfections with *Ascaris*, whipworms and hookworms as well as schistosomes are common in endemic areas, especially among children in regions with poor socioeconomic status, low hygiene and poor sanitation (Pullan et al., 2008).

### 6.1 Host tissues affected by helminth-helminth coinfections

The life cycles of STHs differ significantly between the major species. The larval stages of *Ascaris* and hookworm species undergo extensive migration through the body. *Ascaris* larvae hatch in the intestine followed by migration *via* the liver and lung before being coughed up and swallowed. Upon maturation, the worms reproduce in the small intestine (**Figure 1**). Thus, in *Ascaris* infection the small intestine, the liver and the lungs are dominantly affected and inflammation as well as cellular remodeling and modulation are detectable (Midha et al., 2021). In contrast, hookworm larvae invade the host through the skin and reach the blood circulation which they leave in the lungs (**Figure 1**). Like *Ascaris* larvae, hookworm larvae are then coughed up and swallowed, reaching finally the small intestine. Similar to *Ascaris*, hookworm larvae migrate through the body and thus affect the skin, blood circulation, the lungs and the small intestine of infected hosts. In comparison, whipworm infections in humans occur when the eggs are ingested, as is the case with *Ascaris* infections. The larvae hatch in the small intestine and adult worms establish and dwell in the large intestine (**Figure 1**). Whipworms do not show a body migration, the organ primarily affected is the intestine. However, intriguingly, intestinal worms without body migration also do affect the entire mucosal system and anti-helminth effector and memory responses are clearly detectable in the lungs (Yordanova et al., 2022).

### 6.2 Influence of helminth-helminth coinfections on the host immune response

Although multiple helminths are detected in infected individuals (Donohue et al., 2019), data on host immunological consequences of helminth coinfections are sparse. Some studies in animal models with poly-helminth infections indicate significant immunological effects of the second worm infection. For instance, in pigs coinfected with *T. suis* and *Oesophagostomum dentatum* the *O. dentatum*-specific IgG1 and IgG2 levels in poly-infected pigs were two-fold higher than in *O. dentatum* mono-infected pigs (Andreasen et al., 2015). Thereby, the *Trichuris infection* enhanced the typically weak response induced by *O. dendatum.* These data were underpinned in addition by a stronger Th2 response, measured by parasite-specific IL-4 secreting cells in PBMCs and associated gene expression (*IL-13, ARG1, CCL11*) at the site of infection as well as by peripheral eosinophil counts. Thus, one helminth infection overrides the coinfecting second worm infection (**Figure 4D**). A prominent effect was also demonstrated in a coinfection with the nematode *H. polygyrus* and the trematode *S. mansoni* in mice. Here, a pre-infection with *H. polygyrus* resulted in a marked reduction of hepatic egg-induced granulomatous inflammation associated with dampened proinflammatory cytokines in mice coinfected with *S. mansoni* (Bazzone et al., 2008). Thereby a dominant Th2-polarized environment induced by one infection ameliorated the immunopathology induced by the second helminth infection (**Figure 4D**). Another study in mice using coinfections of *T. muris* and *H. polygyrus* showed that coinfections are capable of changing susceptibility to infection (Colombo et al., 2022). The authors observed a complete impairment of *T. muris* expulsion in immunocompetent mice when coinfected with *H. polygyrus*. Interestingly, this drastic effect was not coupled with a change in parasite-specific cytokine production in draining lymph nodes or mucosal barrier immune responses in coinfected mice. However, changed susceptibility did also not impair immunization-induced immunity (**Figure 4D**) which was attributed to helminth excretory-secretory products (ES products) (Colombo et al., 2022).

In conclusion, multiple helminth infections are common and have significant consequences on the outcome of each infection (**Figure 4D**): i) one helminth infection overrules the immune response to the second infection; ii) the temporal sequence of the respective infection is important for the immunopathological consequences; iii) host susceptibility or resistance can be changed by the coinfecting helminth but is independent of immunization-induced immunity.

## 7 Therapeutic interventions in STH coinfections

The main intervention to control STH in endemic regions is preventive chemotherapy recommended by the World Health Organization (WHO), defined as the regular administration of anthelmintics on a large scale to populations at risk (World Health Organization, 2022). There are different anthelmintics recommended by the WHO for the treatment of STHs: the two benzimidazoles, mebendazole (MBD) and albendazole (ALB), whereby ivermectin was added recently for the control of *S. stercoralis* (World Health Organization, 2023). To reduce morbidity from STH infections, particularly in preschool and school-aged children, but also in high-risk groups (i.e., pregnant women, lactating women, or adults in high-risk occupations), the WHO has established guidelines for large-scale preventive chemotherapy through mass drug administration (MDA) programs (World Health Organization, 2023). MBD and ALB are widely used in MDA campaigns in endemic countries. When administered orally once, both drugs are highly effective against *A. lumbricoides* but significantly less effective against *T. trichiura*. ALB, on the other hand, has better efficacy against hookworms compared to MBD (Moser et al., 2019). However, regular treatment is needed because eggs or larvae that remain in the environment for many months can be the cause of reinfection (Midha et al., 2021). In addition, a network meta-analysis revealed an alarming decline in ALB and MBD efficacy, which may indicate the development of anthelmintic resistance (Moser et al., 2018).

Where STH and schistosomiasis coincide, the WHO recommends that praziquantel (for schistosomiasis) and MBD/ALB can be safely administered together, and this is being done, particularly in Sub-Saharan Africa, where most of these infections occur (World Health Organization, 2022).

For other coinfections with STH, including viral and bacterial coinfections, there are no clear guidelines for therapeutic interventions other than treating each infection once it is diagnosed. There are conflicting results on the effect of deworming in HIV and STH coinfected individuals receiving concomitant antiretroviral therapy. A study in Tanzania found a low prevalence of intestinal helminths in HIV-infected patients compared with HIV-negative participants, presumably because the HIV-infected participants received anthelmintics for prophylaxis (Mwambete et al., 2010). Studies have also reported lower viral loads and higher CD4+ T cell counts in HIV-STH coinfected individuals treated with anthelmintics (Wolday et al., 2002; Ivan et al., 2015; Means et al., 2016), while other studies have found no effect (Lawn et al., 2000; Modjarrad et al., 2005; Hosseinipour et al., 2007). Therefore, further long-term studies are needed to determine the safety and benefit of preventive chemotherapy to control STH in HIV-positive patients.

When STH and malaria coinfections occur, each disease is often treated with a different strategy. For STH, preventive chemotherapy is used; for malaria, intermittent preventive treatment (IPT) and seasonal malaria chemoprevention (SMC) are used. Artemisinin-based combination therapies are currently the most effective and widely used agents for malaria control. SMC is used in endemic malaria areas during the period of highest malaria risk and involves the administration of the full dose of antimalarials at monthly intervals to maintain drug concentrations in the blood (Coldiron et al., 2017). Efforts are underway to integrate a unified strategy to control co-occurring diseases such as malaria and helminths, and evidence suggests that intermittent preventive malaria treatment and deworming reduces the prevalence of anemia and leads to improved cognitive skills in school-aged children (Opoku et al., 2016; Afolabi et al., 2022). Even with disease-specific approaches, some studies have reported benefits beyond the targeted disease. For example, quarterly administration of ALB in children older than 5 years resulted in a lower incidence of clinical malaria (Ndibazza et al., 2012). However, the results of a number of studies are contradictory. For example, a study in Madagascar found a significant increase in *P. falciparum* parasitemia in children aged ≥15 years treated with the anthelmintic levamisole for two months compared with negative controls (Brutus et al., 2006). Several other studies have found no effect of deworming on malaria (Kirwan et al., 2010; Wiria et al., 2013; Kepha et al., 2016). Despite some of these conflicting results, there is a consensus that intensive deworming does not alter the risk of malaria in school-aged children.

However, in addition to the WHO recommendations to treat STH and schistosome coinfections accordingly, general guidelines for other coinfections are lacking. As mentioned above, helminth coinfections can influence the immune response in a way that favors the development and spread of co-occurring microbial infections. Hence, it should follow that treatment of the microbial coinfection might be more promising if the STH infection has been eliminated before or, at the latest, in parallel if there are no concerns about deleterious drug interactions.

## 8 Outstanding questions and future directions

STHs can change host immunity both locally at the site of infection and remotely in tissues that are not parasitized. Therefore, the interactions are very complex and seem to depend largely on the context and biology of the coinfection in a bidirectional manner. Pioneering studies in rodent models provided important mechanistic insights into different coinfection scenarios, however, limitations of the translatability of the rodent models are obvious, since no natural infections with STHs relevant for humans occur in them. Here, the pig represents a valuable human-relevant model for the study of infectious diseases (Schlosser et al., 2014; Ebner et al., 2017; Schmidt et al., 2020), since closely related STH species with even zoonotic potential (e.g. *T. suis*, *A. suum*) and viruses (e.g. HEV, influenza A virus) or bacterial species (e.g. *S.* Typhimurium), relevant to humans, occur naturally in the pig. In the future, more complex *in vitro* systems integrating stromal and immune cells and microengineering technologies with organoids offer great potential to further advance animal-based research on STHs and relevant coinfecting pathogens while expanding knowledge of mechanistic processes.

Despite the ameliorative effect of some coinfections with enteric helminths in a few studies (Chowaniec et al., 1972; McFarlane et al., 2017; Rolot et al., 2018), some experimental studies have shown that the timing of parasite infection and host age can be crucial for the outcome of concurrent microbial disease (Furze et al., 2006; Hussain et al., 2023) and that prior tissue damage from larval migration may also be particularly important (Wescott and Todd, 1966; Gazzinelli-Guimarães et al., 2017). Nevertheless, only few studies that addressed interactions between helminths and other pathogens examined the effects of coinfection on the worm’s life cycle and related host immune responses, such as modulation of larval migration, adult worm establishment, and persistence in the gut (Coomes et al., 2015; Ahmed et al., 2017). The question of how disruption of the epithelial barrier by coinfecting intestinal pathogens might drive or even hinder the establishment of helminth infection in the intestine and what consequences this has for the anti-STH immune response is therefore only beginning to be elucidated and requires further investigation. Whether altered hepatic immune responses and profibrotic mechanisms during coinfection have an impact on larval migration and subsequent intestinal settlement of adult worms remains to be elucidated as well. As little is known to date, targeted studies investigating the effect of therapeutic interventions in STH and microbial coinfections are urgently needed to develop consensus guidelines for the treatment of co-occurring infections.

However, efforts to reduce helminth infections through preventive chemotherapy may have spillover effects on other infections or even metabolic disorders, which in turn can be beneficial or detrimental to the host’s health. In this context, it is therefore controversial to discuss whether certain susceptibilities to infectious diseases and associated morbidities in populations underscore the need to restore the lost biodiversity of eukaryotic symbionts such as helminths.

## Author contributions

Conceptualization, JS-B, AM, SH. Writing — original draft preparation, JS-B, AM, SR, SH, RM, GG, EN, DN. Writing — review and editing, JS-B, AM, SR, SH, RM, GG, EN, DN. All authors contributed to the article and approved the submitted version.
